# Impact of breastfeeding interventions among United States minority women on breastfeeding outcomes: a systematic review

**DOI:** 10.1186/s12939-021-01388-4

**Published:** 2021-03-06

**Authors:** Sofia Segura-Pérez, Amber Hromi-Fiedler, Misikir Adnew, Kate Nyhan, Rafael Pérez-Escamilla

**Affiliations:** 1 Hispanic Health Council, 175 Main Street, Hartford, CT 06106 USA; 2 Yale School of Public Health, 135 College Street, New Haven, CT 06510 USA; 3 Harvey Cushing/John Hay Whitney Medical Library, Yale School of Public Health, New Haven, USA

**Keywords:** Breastfeeding, Ethnic/racial minorities, Policy, Socioecological model

## Abstract

**Background:**

In the U.S., strong ethnic/racial, socioeconomic, demographic, and geographic breastfeeding (BF) inequities persist, and African American and Hispanic women are less likely to meet their breastfeeding goals compared to White women. This systematic review (SR) was designed to answer the question: What is the impact of breastfeeding interventions targeting ethnic/racial minority women in the U.S. on improving BF initiation, duration and exclusivity rates?

**Methods:**

The SR was conducted following the Institute of Medicine Guidelines and the Preferred Reporting Items for Systematic Reviews and Meta-Analyses checklist. The study protocol was developed and registered a priori in PROSPERO (ID#CRD42020177764). The electronical databases searched was MEDLINE All (Ovid). Search strategies were led by the team’s expert public health librarian using both controlled vocabulary and free text queries and were tested against a validated set of relevant papers included in existing reviews. The GRADE methodology was used to assess the quality of the studies.

**Results:**

We included 60 studies that had randomized (*n* = 25), observational (*n* = 24), quasi-experimental (*n* = 9), or cross-sectional (n = 2) designs. The studies focused on populations that were multi-ethnic/racial (*n* = 22), only Hispanic (n = 24), only Black (*n* = 13), and only American Indian (n = 1). The study interventions were classified following the socioecological model: macrosystem/policy level (*n* = 6); community level (n=51), which included healthcare organizations (*n* = 34), The Special Supplemental Nutrition Program for Women, Infants, and Children (WIC) (*n* = 9), and community organizations/public health institutions (*n* = 8); and  interpersonal level (n = 3).

**Conclusions:**

Policy and community level interventions delivered through WIC, healthcare facilities, and community agencies) are likely to improve BF outcomes among women of color. The combination of interventions at different levels of the socioecological model has not been studied among minority women in the U.S. Implementation science research is needed to learn how best to scale up and sustain effective BF interventions, taking into account the needs and wants of minority women. Thus, it is strongly recommended  to conduct large scale implementation research studies addressesing how to strengthen the different health and social environments surrounding women of color in the U.S. to improving their BF outcomes.

**Supplementary Information:**

The online version contains supplementary material available at 10.1186/s12939-021-01388-4.

**Table:** 

This article is a part of the Interventions and policy approaches to promote equity in breastfeeding collection, guest-edited by Rafael Pérez-Escamilla, PhD and Mireya Vilar-Compte, PhD	

## Background

A constellation of short and long-term health benefits of breastfeeding (BF) for mothers and children have been well documented [1–3]. Breastfed compared to non-breastfed infants have lower rates of infectious diseases, childhood obesity, and improved cognitive outcomes [4–6]. Additionally, BF protects mothers against breast and ovarian cancers, type 2 diabetes, and cardiovascular disease [3, 7]. These benefits increase with BF exclusivity during the first 6 months of life and longer duration of any BF [3, 4, 7].

In the United States of America (U.S.), the Healthy People Initiative (HP) establishes evidence-based goals with the purpose of monitoring and improving the health of all Americans [8]. Increasing the proportion of breastfed infants is one of the Healthy People 2020 (HP2020) goals to improve maternal, infant, and child health nationally [8]. Five specific BF indicators and corresponding targets are used to monitor the progress made towards reaching this HP2020 goal: BF initiation (81.9%), any BF at 6 months (60.6%), exclusive BF (EBF) at 3 months (46.2%), EBF at 6 months (25.5%), and continued BF at 12 months (34.1%) [9]. The Centers for Disease Control (CDC) Breastfeeding Report Card, produced every two years, reports on these indicators using data from the annual National Immunization Survey (NIS). Findings from the 2020 Report Card, which used National Immunization Survey (NIS) data from infants born in 2017 [10], showed improvements in all HP2020 BF targets (compared to the 2018 Report Card) and almost all targets were reached or surpassed [11]. Indeed, most mothers initiated BF (84.1%), more than half of mothers breastfed for 6 months (58.3%), 35.3% of mothers breastfed through 12 months, almost half of mothers did EBF for 3 months (46.9%), and one quarter of mothers did EBF for 6 months (25.6%). When BF rates from the 2020 report card were compared with those from the 2011 report card, the time at which the HP 2020 goals were first assessed, it was found that BF initiation, duration, and exclusivity rates in the U.S. had improved significantly over the last 10 years [12]. However, the 2017 NIS data showed that BF inequities continue to exist. Non-Hispanic Black infants continued to have the lowest prevalence for having ever been breastfed (73.7%) compared with non-Hispanic White infants (86.7%) as well as all the other racial groups (Hispanic 84.1%, Asian 90%, Hawaiian/Pacific Islander 85.2%, American Indian (AI)/Alaska Native (AN) 80.7%) [13]. Non-Hispanic Black infants also had lower BF duration and exclusivity rates for all HP2020 BF indicators when compared with non-Hispanic Whites, indicating that this racial inequity in BF persists [14–16]. Among Hispanics, while they had similar BF initiation rates as non-Hispanic Whites, they had significantly lower rates for BF duration and exclusivity. Only non-Hispanic White and Asian women achieved all the targeted indicators set by HP2020. Although BF duration and exclusivity data could not be calculated for Hawaiian/Pacific Islander and AI/AN using the 2017 NIS data [13], an article presenting earlier NIS data from 2013 showed that AI/AN and Hawaiian/Pacific Islander women were below the HP2020 BF targets, and had significantly lower BF initiation, exclusivity, and duration rates than their White counterparts [17].

In the U.S., strong and persistent socioeconomic, demographic, and geographic BF inequities also persist. The 2017 NIS data demonstrated lower BF initiation rates among women with less than a high school education (73.6%), younger than 20 years old (74%), with incomes below the poverty level (76.6%), who were single mothers (75.1%), and living in non-metropolitan areas (77.5%) [13]. These findings are important for understanding BF inequities among minority women who, in large part due to social inequities, are strongly affected by poverty, low levels of education, vulnerable family structures, and geographical segregation. Disadvantages with regards to these social determinants of health pose many BF challenges, including lack of access to quality BF education, social and lactation management support, maternity protection benefits, and culturally appropriate health care services [18–21].

Among BF women, the early introduction of formula has been linked with shorter BF duration and exclusivity [22, 23]. For this reason, it is concerning that the 2020 BF Report Card shows that 19.2% of infants that were ever breastfed received formula before 2 days of age, which increased from rates reported in the 2018 BF Report Card (17.2%). However, a recently published analysis based on NIS data from 2009 to 2015 and broken down by race/ethnicity among BF infants, shows that the prevalence of formula supplementation at < 2 days in 2015 was higher among Hispanic (23.2%), Black (20.9%), and Asian (21.4%) infants compared with non-Hispanic Whites (12.7%) and the national rate (17.2%) [24]. Furthermore, formula supplementation rates at < 2 weeks postpartum were higher among Hispanics (27.0%), Blacks (25.3%), and Asians (25.5%) compared with Whites (17.5%) and the national average (21.7%) [25].

BF ethnic/racial inequities are a major concern because of the inability for most socio-economically vulnerable women to not be able to BF for as long as is recommended, which can be considered as a violation of their right to make the best decision on how to feed their infants [26, 27]. Furthermore, this inequity leads to hundreds of millions of dollars lost in healthcare expenditures and work productivity since the health of women and their children is put at risk as a result of not being able to  BF as recommended [28].

### Objectives

The current BF situation in the U.S. calls for improving investments in protecting, promoting, and supporting BF among minority women. For this reason, it is important to identify effective interventions that have the potential to be scaled up to continue improving BF while at that same time reduce racial/ethnic BF inequities. Thus, the main goals of this systematic review (SR) are to: (a) integrate the literature on interventions conducted in the U.S. among minority pregnant or postpartum women designed to improve BF outcomes (BF initiation, duration, and exclusivity), (b) map them to the socioecological model [29, 30] and (c) assess the interventions’ potential for scaling up following the Breastfeeding Gear Model (BFGM) framework (see Table 1) [31].

**Table 1:** The Breastfeeding Gear Model

The evidence-based Breastfeeding Gear Model (BFGM) posits that the engine needed by countries to successfully scale up and sustain breastfeeding programs at the national level includes 7 peripheral gears and a master coordinating gear [31]. Evidence-informed **advocacy** is needed to generate the **political will** that results in **legislation** leading to policies with earmarked **resources** for the effective protection, promotion and support of breastfeeding. These resources are essential for building and sustaining the workforce capacity through sound education and training and for the proper implementation of facility (e.g., Baby Friendly Hospitals) and community based (e.g. breastfeeding counseling) programs (**training and implementation** gear). **Social marketing** through behavior change communication campaigns that address society at large are needed to educate and elicit support for breastfeeding mothers including family members, friends, health care providers, and decision makers. Implementation **research and evaluation** is needed to ensure that scaling up bottle neck are identified and addressed on time for long term success and sustainability. The central master or **coordination** gear is typically conformed by a national breastfeeding coordinating entity that needs to be well funded and empowered to monitor goals and lead the intersectoral coordination needed to ensure that the complex breastfeeding system work. The BFGM has been operationalized into the Becoming Breastfeeding Friendly initiative (BBF), a policy tool-box for decision makers interested in scaling up their breastfeeding programs at the national level using evidence of strengths and weaknesses in each of the gears of the BFGM. BBF includes metrics and how to set up a policy development process through an intersectoral committee where civil society organization and international agencies are also represented [123, 124]. BBF has now been successfully applied in 8 countries across 5 world regions; Germany, Ghana, England, Mexico, Myanmar, Samoa, Scotland, and Wales (Pérez-Escamilla R. Becoming Breastfeeding Friendly, Yale School of Public Health. www.bbf.yale.edu (accessed October 27, 2020).	

The primary question that this systematic review answers is: What is the impact of breastfeeding interventions targeting minority women in the U.S. on improving BF initiation, duration, and exclusivity rates among this population? This SR is needed because previous ones focused on single ethnic/racial groups (i.e. African American women [32], Hispanic [33], and Native Hawaiians and Pacific Islander women [34]) or were conducted over a decade ago [35]. Furthermore, except for one conducted focusing on an African-American population [32], none of these reviews mapped findings through the lens of the socioecological model nor did they examine the scaling up potential based on systems thinking approaches.

## Methods

This SR was conducted following the Institute of Medicine Guidelines [36] and the Preferred Reporting Items for Systematic Reviews and Meta-Analyses checklist [37]. The study protocol was developed and registered a priori in PROSPERO (ID#CRD42020177764).

### Literature search strategy

We searched the database MEDLINE on the Ovid platform. Ovid MEDLINE ALL contains records from 1946 through the most recent daily update. It includes not only MEDLINE records but also epub ahead of print, in-process, and other non-indexed citations. Although in our protocol, we anticipated searching three other databases: CINAHL (Ebsco), Embase (Ovid), Web of Science Core Collection (as licensed at Yale), and PsycINFO (Ovid); we did not do so because of the comprehensiveness of the MEDLINE ALL search in the context of limited resources.

The sensitive search strategy was developed by a medical librarian (KN), tested against validation articles previously identified by the authors, and peer reviewed by an independent medical librarian using the Peer Review Electronic Search Strategies (PRESS) guidelines [38]. The full search strategy is provided in an online supplementary material 1. It includes controlled vocabulary and keyword searches for the concepts of breastfeeding and minority populations. A validated hedge for health equity papers was considered, but after testing against our validation articles, we preferred an approach that focused on search terms for minority groups and socio-economic status, rather than the longer list of health equity terms articulated by Prady [39].

Articles published before 2009 were not retrieved for screening because of changes in breastfeeding policy recommendations at that time. Articles indexed with the animal label were not retrieved for screening unless they were also indexed with the human label. Articles indexed with a geographic subject heading were not retrieved for screening unless their geographic label was potentially related to the United States; articles with no subject indexing or United States subject indexing were retrieved and screening. This approach increased specificity without reducing sensitivity and allowed us to operationalize the minority population concept of the search with many potentially relevant terms.

The MEDLINE records retrieved by the search on April 17, 2020 were uploaded to Covidence, which identified all duplicate records.

### Inclusion and exclusion criteria

Quantitative studies were evaluated for eligibility based on the following selection criteria: (a) early defined BF interventions or exposures targeting ethnic/racial minority women (i.e., at least 70% in the sample, or reporting analyses broken down by ethnic/racial group) living in the U.S. or any of its territories and conducted during pregnancy, perinatally and/or during the post-partum period; (b) targeting healthy pregnant women with a full-term singleton healthy baby and/or postpartum women; and (c) reporting on one or more of the following BF outcomes: BF initiation, EBF during the first 6 months or any BF within the first year of the infant’s life. Studies were excluded from the SR if they (a) did not target minority women (i.e. if less than 70% of the sample were minority women) or did not conduct analyses of BF outcomes by ethnic/racial group; (b) were not conducted in the U.S. or any of its territories; (c) targeted or included > 10% of mothers classified as having a high risk pregnancy; (d) focused on women carrying more than one baby or having a preterm baby; or (e) were published before January 2009.

### Study selection process

The records screening process was done using Covidence online software [40]. To ensure consistency across study, three researchers (SSP, AHF, MA) independently screened the first 200 abstracts and screening differences were resolved through a consensus process facilitated by two co-authors (KN and RPE). Then, the operationalization of the inclusion criteria was finalized. The remaining abstracts were divided and independently reviewed by the three co-authors (SSP, AHF, MA). Consensus was reached on the papers that were eligible for a full text review (*n* = 315). The lead author (SSP) reviewed all full text articles while the two co-authors (AHF, MA) each independently reviewed half to ensure each article was reviewed twice. Discrepancies among SSP and AHF or MA were resolved through a consensus group approach as well as with support from the senior author (RPE).

### Data extraction

Data extraction was led by two reviewers (SSP and AHF) with support from two co-authors (MA and RPE). A total of 60 studies met final eligibility and data were extracted for study aim, design, recruitment procedure, methods, intervention characteristics (level of socioecological model addressed), description of intervention or exposure, outcomes, results, and conclusion. Included studies were then organized and presented by socio-ecological model level [29, 30]. 

### Quality assessment and risk of bias

The Grades of Recommendation, Assessment, Development, and Evaluation (GRADE) was used to assess the quality of evidence. GRADE is a study quality assessment approach that follows a standardized method, it is based on an in-depth assessment of different study design risk of bias parameters to classify the quality of evidence as either high, moderate, low or very low. GRADE was used to assess the quality of randomized controlled trials (RCT), quasi-experimental studies, or observational studies according to prespecified criteria [41]. The risk of bias for RCT’s was based on the following criteria: randomization procedures, lack of blinding, lack of allocation concealment, incomplete accounting of participants and outcome events. All RCTs were initially ranked as high quality and then could be downgraded based on the extent of the risk of bias. Observational studies were initially ranked as being of low quality but could be upgraded or downgraded based on risks of bias (which focused on measurement of exposures and outcomes, adjustment of confounding factors, statistical analysis, and attrition rate). A similar approach was used for quasi-experimental studies but grading also took into account the appropriateness of the comparison group for addressing confounding. Each study was graded by at least two of the authors (SSP, RPE, AHF). Prior to grading we ensured that the three co-authors were standardized against each other on assigning a quality score to each study based on the risk of bias assessment. The lead author first graded 10 studies and the two other co-authors reviewed the same 10 (5 studies each). The team then met to compare and confirm their consensus on grading scores across studies.

## Results

### Literature search

**Fig. 1:**
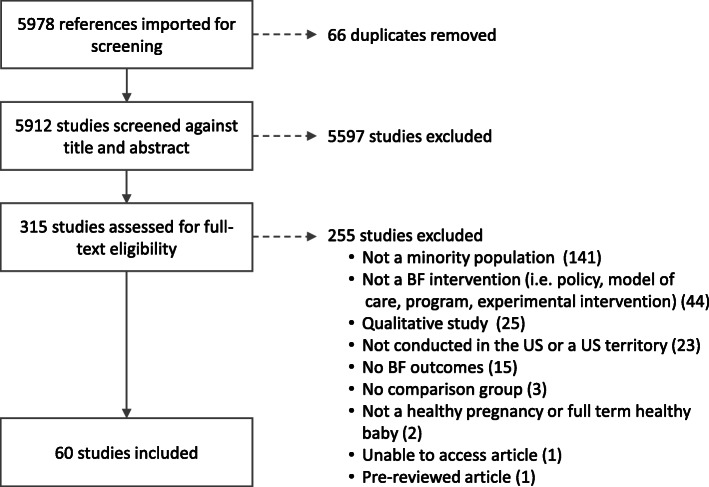
Systematic review PRISMA flow chart; breastfeeding interventions among women of color in the U.S.

As shown in the PRISMA flow chart (Fig. 1) a total of 5978 articles were initially identified. Once 66 duplicates were removed, the titles and abstracts of the remaining 5912 articles were screened for SR inclusion and exclusion criteria. As a result, 5597 were eliminated from further consideration. The remaining 315 articles were selected for full-text screening. Of these, 243 articles were excluded in the first phase of full-text screening and subsequently 12 additional articles were excluded while conducting data extraction. Thus, a total of 60 studies met the inclusion criteria for this review. The detailed data extraction information for each study can be found in online supplementary material 2.

### Study characteristics

The final set of 60 articles were based on studies with randomized (*n* = 25), observational (*n* = 24), quasi-experimental (*n* = 9) and cross-sectional (n = 2) designs. The articles focused in populations that were multi-ethnic/racial (*n* = 22), only Hispanic (n = 24), only Black (*n* = 13), and only American Indian (n = 1).

**Fig. 2:**
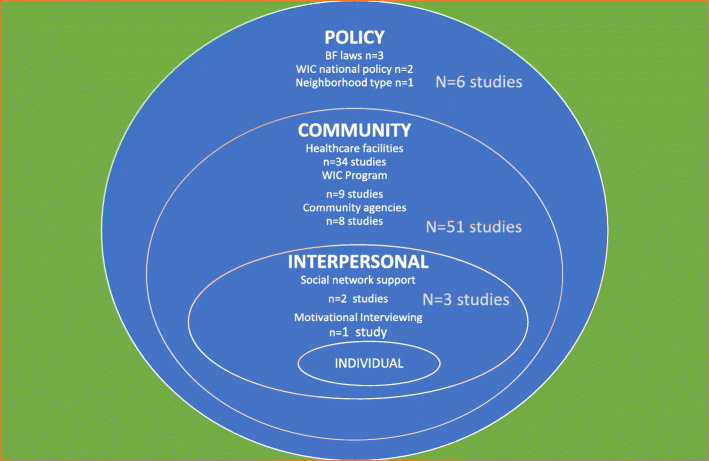
Breastfeeding studies identified for systematic review by socioecological model level among women of color in the U.S.

Since BF decision and practices are affected from the proximal to the distal environments surrounding women, infants, and their families, the socioecological framework was used to examine the relationship between BF intervention/exposures and BF outcomes among minority populations. The level of the socioecological at which the intervention was placed was chosen based on the institution that would be expected to manage the intervention if it were to be scaled up. For example, Baby Friendly Hospital Initiative (BFHI) interventions are delivered at the individual level and can even be linked to home-based peer counseling services through Step 10 of the BFHI. However, at the end of the day, the delivery of BFHI depends on health care facilities. Hence, BFHI studies were classified at the community level of the socioecological model under health care facilities. Following this reasoning, the articles included in this systematic review (SR) were classified as part of  the a) macrosystem/policy level (*n* = 6); b) community level (including healthcare organizations (*n* = 34), WIC program (*n* = 9) and community organization or public health institution (*n* = 8) ); or c)  interpersonal level (n = 3) (Fig. 2). Thus, findings of this review are synthesized at these levels of the socioecological framework.

#### Macrosystem/policy level

The six studies identified as part of the macrosystem/policy level included analyses of the impact of BF policies among racial and ethnic groups. Four had pre/post observational designs, one retrospective and one was quasi-experimental. Their quality was graded as moderate (*n* = 1) or low (*n* = 5) (Table 2).

**Table 2:** Policy level breastfeeding interventions among women of color in the U.S.

Study	Study Design/Data collection	Study Population	Intervention/Exposure	Outcomes/Results	Grade
Kapinos et al. [42] National data	**Design:** Pre/post observational **Data:** 2009–2014 National Vital Statistics System	*N* = 17,985,584 births; H: 33.42%; W: 77.04%; AA: 15.72%; AI: 0.99%; A/PI: 6.47%	Before/After Affordable Care Act (ACA 2009 versus 2013, 2009 versus 2014)	ACA implementation associated with AA & AI/AN mothers being 1–2% points more likely than W women to initiate BF.	Low
Hawkins et al. [43] PRAMS data	**Design:** Quasi-experimental **Data:**2000 & 2008 Pregnancy Risk Assessment Monitoring System (PRAMS) 32 states.	*N* = 349,780 mothers; 2000–2008: W:55.7–53.3%; H:12.4–15.1%; AA:18–16.4%; Other:13.6–15%	Before/After BF state laws: 1) worksite breaktime and private space for BF 2) BF anywhere	H mothers had 5.8%-point increase in BF initiation in states with worksite laws. AA mothers had 5.6%-point increase in BF initiation in states with laws for BF in any location.	Low
Smith-Gagen et al. [44] National data	**Design**: Pre/Post observational **Data:** 2003–2010 U.S. NHANES linked to Policy data	*N* = 3132; AA = 609, H = 1078 W = 1445	Before/After 8 BF protection laws^a^	At 6 mo AA were half as likely to BF for jury duty exemption; H were more likely to BF with break time from work /pumps access when compared with W.	Low
Li et al. [45] National data	**Design:** Pre/Post Observational **Data:**2000–2014 NHANES data of WIC eligible < 5 y old children.	2000–2008: *N* = 2770; W = 25.31%; AA = 22.20%; H = 45.92%. 2009–2014: *N* = 1538; W = 22.63%; AA = 27.63%; H = 39.92%	WIC eligible participants and non-participants before and after 2009 WIC package.	2009–2014 BF initiation rates improved for WIC participants, and the differences among non-participant became non significant as it was in 2000/2008. No effect on BF duration.	Moderate
Langellier et al. [46] WIC survey	**Design:** Pre/post observational **Data:** WIC LA County Survey	*N* = 5524; in 2005 84.9% H; in 2011 80% H	2009 WIC Package revisions; 2011 vs. 2005 and 2008	In 2011 BF initiation, EBF for 3 mo., and EBF at 6 mo. significantly increased. No effect on Any BF at 3&6 mo	Low
Yourkavitch et al. [47] Data sets	**Design:** Retrospective **Data:** New Jersey birth certificate and medical records data	EBF sample, *N* = 26,287: W = 60%;AA = 10%;H = 20%; A/PI = 10%. Any BF, *N* = 54,117:W = 43%;AA = 13%;H = 33%; A/PI = 12%	Neighborhood (NBHD) disadvantage versus affluence	EBF decreased as NBHD disadvantage increased only for Asian, AA, and H. EBF increased as NBHD affluence increased for H and W. Any BF decreased as NBHD disadvantage increased among H and W.	Low

^a^8 laws: 1) BF exempt from indecency laws, 2) laws allowing women to BF in public/private locations, 3) laws to exempt BF women from jury duty, 4) laws to encourage BF awareness campaigns, 5) laws allowing a reasonable unpaid breaktime from work to express milk, 6) laws requiring a sanitary space at work to express milk, 7) enforcement of workplace pumping laws and 8) enforcements of public BF laws. Abbreviations: *AA* African American; *A/ PI* Asian/ Pacific Islander; *H* Hispanic; *W* White; *NBHD* Neighborhood; *WIC*, Special Supplemental Nutrition Program for Women, Infants and Children; *NHANES* National Health and Nutrition Examination Survey; *BF* breastfeeding; *EBF* Exclusive breastfeeding

Three studies evaluated the association between BF promoting laws and BF initiation and duration (one at federal policy level, two at state level) [42–44]. Two reported positive outcomes on BF practices, especially among minority women [42, 43]. An observational study by Kapinos et al. [42] focused on the influence of the mandated coverage of lactation support services under the Affordable Care Act (ACA). After ACA was enacted, Black/African-American (AA) women and American Indian/Alaska Natives (AI/AN) were more likely to initiate BF (by 1–2 and 1%, respectively) compared to their non-Hispanic White counterparts. A state-level policy study by Hawkins et al. [43] assessed the influence of two maternity protection laws on BF: a) employers providing breaks and space for BF to employees and b) BF being permitted in any public or private location. Pregnancy Risk Assessment Monitoring System (PRAMS) weighted data (2000–2008) from thirty-two states was used to assess BF initiation and duration before and after policy enactment. For each law, each mother received a code of *yes* if the law had been in place in the 6 months prior to the delivery of the newborn or *no* if otherwise, based on the law’s effective date. Findings showed that BF initiation rates were significantly higher in states with laws in place that provided BF break time and private space by employers. While Hispanics had a 5.8 percentage point increase in BF initiation in states where the new law was enacted, this association was not seen among Black mothers. However, a 5.6 percentage point increase on BF initiation was seen among Black women within states with new laws permitting BF in any location.

A pre/post observational study by Smith-Gagen et al. [44] used 2003 to 2010 data from the National Health and Nutrition Examination Survey (NHANES) and assessed the impact of eight BF supportive laws enacted at the state level. The laws examined were: 1) exempting BF from public indecency, 2) allowing women to breastfeed in public and private locations, 3) exempting BF women from jury duty, 4) encouraging BF awareness campaigns, 5) allowing a reasonable unpaid breaktime from work for BF mothers to express milk, 6) requiring a sanitary space at work for BF mothers to express milk, 7) permitting workplace pumping, and 8) permitting public BF. Analyses, which were stratified by race/ethnicity, found no differences in ever BF rates by law between racial/ethnic minority groups and non-Hispanic Whites. When examining BF duration, Mexican Americans mothers, compared to non-Hispanic Whites, were 30% more likely to breastfeed for at least 6 months in states providing break time from work (AOR, 1.3; 95% CI, 1.0–1.8). Conversely, compared with non-Hispanic Whites, Black mothers were less likely to breastfeed for at least 6 months in areas with laws protecting break time at work places (AOR, 0.6; 95% CI, 0.5–0.8), areas providing an exemption for jury duty (AOR, 0.6; 95% CI, 0.4–0.9), and areas with pumping law enforcement (AOR, 0.6; 95% CI, 0.5–0.8).

Two observational studies examined the effect of the 2009 Supplemental Nutrition Program for Women, Infants and Children’s (WIC’s) package on BF practices [45, 46]. Li et al. [45] compared two NHANES cross-sectional cohorts (2000–2008 versus 2009–2014) before and after the implementation of the new 2009 WIC package. Among WIC participants, improvements were seen in BF initiation between 2009 and 2014 but not in BF duration at 6 months. A study by Langellier et al. [46] used a pre/post design with data from the Los Angeles WIC triennial survey (2005, 2008 and 2011) to assess the effect of the 2009 WIC package on BF outcomes among WIC participants in Los Angeles County. Findings showed that participants who received the new food package had 2.2 times the odds of initiating BF (*P* < .001), 1.7 times the odds of exclusively BF at 3 months (P < .001), and 3.1 times the odds of exclusively BF through 6 months (P < .001). No effects were seen on any BF rates at 3 and 6 months.

The last study, a retrospective observational design by Yourkavitch et al. [47], assessed the relationship between living in neighborhoods with different levels of disadvantage and affluence and BF outcomes. The study used 2006 birth certificate data linked to census tracks in New Jersey. Findings showed that the probability of EBF was lower as neighborhood disadvantage increased for all populations (Asian, Black, Hispanic) except non-Hispanic White women. As neighborhood affluence improved, the probability of EBF increased among Hispanic and non-Hispanic White women only while the odds of any BF increased for all racial groups except Hispanic women.

These findings suggest that BF protection laws such as the provision of work breaks and space by employers seem to benefit Hispanic women the most, and laws protecting BF in private and public places protect Black women more. More needs to be understood about the effect of these laws among the different racial/ ethnic minorities. Changes in federal food assistance policies may also positively influence BF outcomes among racial/ethnic minority populations as intended by the changes made to the WIC BF packages in 2009 to incentivize EBF. However, effect sizes tended to be small implying that, in addition to policies, the delivery of BF programs at the community level is needed. Most studies were observational of moderate (*n* = 1), and low (*n* = 4) quality, and the quasi-experimental study was classified as low quality as well. The studies in the organizational/community level of the socioecological model are presented in the following section.

#### Organizational/community level

Most (85%) of the 60 interventions included in this review were conducted at the organizational/community level (*n* = 51). Of these, 33 were implemented at healthcare institutions, 9 were WIC programs, and 9 were based at community maternal and child health programs. Interventions at healthcare institutions are divided into implementation periods: pregnancy (n = 5); in the maternity ward (*n* = 7); pregnancy and post-partum (n = 5); pregnancy, delivery and post-partum (*n* = 6); post-partum [8]. The results are presented based on these implementation periods.

### Healthcare interventions

#### Prenatal

There were five BF studies, all observational designs, conducted primarily during pregnancy [48–52]; three were with women enrolled in the Centering Pregnancy Model (CPM) and had mixed results. The first observational prospective study conducted by Chae et al. [48] in a prenatal New Jersey clinic among low-income, predominantly minority women found that women receiving CPM prenatal care were two times more likely to be BF at their postpartum visit (OR = 2.9, *p* = 0.001) compared with those receiving standard prenatal care. The authors suggest that the social and emotional peer support provided by CPM may explain this finding. By contrast, a second study with a prospective observational design conducted by Robertson et al. [49] in Georgia with Hispanic women receiving CPM prenatal care did not find significant differences on BF outcomes compared with standard prenatal care. However, it should be noted that both studies had high attrition rate. Unexpectedly, a third study with retrospective observational design conducted by Trudnak et al. [50] among Hispanic women receiving CPM prenatal care found that they were more likely to be feeding formula at their newborn visit.

An observational prospective study by Farr et al. [51], conducted with African American women, examined the effect of two 5–10 min iPad prenatal interventions on BF outcomes. One intervention focused on helping the mother identify a supportive BF champion (Champion intervention) while the other provided BF information following a frequently asked questions approach (Positive messaging intervention). The interventions had the most in-hospital impact on EBF rates among women not intending to EBF during pregnancy. However, findings need to be interpreted with caution as the study did not include a comparison group with no intervention and mothers delivered at a Baby Friendly Hospital (BFH). Lastly, an observational study by Louis-Jacques et al. [52] assessed the effect of a prenatal basic educational intervention focused on medications use and lactation among predominantly Hispanic mothers with no contraindications for BF. Findings showed the intervention was not effective in improving BF outcomes since women taking medication were three and four times more likely to mix feed or feed formula at 2–4 week and 6–8 week post-partum respectively.

All studies included in this section were non-experimental with most interventions focusing on various aspects of prenatal care or were simple BF interventions. None of the intervention were adapted or tailored specifically to minority women. Studies were of low (*n* = 3) or very low (*n* = 2) quality (Table 3).

**Table 3:** Community level healthcare facility breastfeeding interventions among women of color in the U.S. : Prenatal

Study/Setting	Study Design/Data collection	Population	Intervention/Exposure	Outcomes/ Results	Grade
Chae et al. [48] Family Medicine Outpatient Clinic	**Design:** Prospective **Data:** 3rd prenatal trimester, 6–8 wks surveys	Center Pregnancy Model (CPM) (*n* = 120); 15% A; 28.3% AA 30% H, 26.7% W Standard Care (SC) (*n* = 221); 16.7% A; 20.3% AA; 34.8% H; 26.7% W	CPM**:** Via health care providers; 10 prenatal and 1 p.p. sessions SC group**:** Individual care	CPM Participants > 2 times more likely to BF at p.p. visit	Low
Robertson et al. [49] Hospital based prenatal clinic	**Design:** Two group, pre/post observational **Data:** prenatal and p.p. surveys	CPM n = 24 H SC n = 25 H	Midwife care to both groups. CPM: first 4 pregnancy months groups met once a month, then biweekly until delivery. SC: Individual prenatal care	NS BF differences at 6 weeks p.p.	Very low
Trudnak et al. [50] Health Department Clinics	**Design**: Retrospective **Data:** Chart review	CPM n = 240 H SC *n* = 240 H	CPM**:** prenatal group care SC: individual care	At 6 wks p.p. CPM participants more likely to FF.	Low
Farr et al. [51] Women’s Health Clinics	**Design**: Observational **Data:** Chart review	Champion iPad app *n* = 132; 96.2% AA Positive messaging iPad app *n* = 111participants. 90.8% AA,	Champion app helped mothers identify supportive BF champion. Positive messaging app offered BF information with picture; FAQ format.	For both apps, proportion of mothers who intended EBF prenatally more likely to EBF in-hospital increased (vs. those not intending EBF)	Very low
Louis-Jacques et al. [52] Prenatal clinics	**Design:** Prospective **Data:** Pre/post; BF survey data at 2–4 and 6–8 wks p.p.	*N* = 121; 63.6% H	Prenatal BF, medication and substance abuse education; via Doulas and IBCLC.	Women using medications at 2–4 and 6–8 weeks p.p. were less likely to EBF and more likely to mix feed or FF vs. mothers not taking medication 2–4 wks p.p. and 6-8 weeks p.p.	Low

Abbreviations: *AA* African American; *A* Asian; *H* Hispanic; *W* White; p.p. postpartum; *CPM* Center Pregnancy Model; *SC* standard care; *NS* not significant; *BF* breastfeeding, *EBF* exclusive breastfeeding; *FF* formula feeding; *IBCLC* Board Certified Lactation Consultant; *app* application

#### Maternity Ward

An observational study conducted by the Communities and Hospitals Advancing Maternity Practices initiative (CHAMPS) between 2014 and 2017 found that the implementation of the Ten Steps from the Baby Friendly Hospital Initiative (BFHI) improved BF outcomes and reduced BF disparities in 4 Southern States. Using evaluation data from 31 of 33 participating hospitals (7 in Louisiana, 17 in Mississippi, 2 in Tennessee, 5 in Texas), findings showed that, as BFHI implementation advanced, the proportion of women who experienced rooming-in and skin-to- skin contact after delivery increased. Breastfeeding initiation rates increased during the same period from 66 to 75% (*p* < 0.05), and EBF rates increased from 34 to 39% (p < 0.05). Furthermore, BF initiation rate inequities for non-Hispanic Black women declined by 9.6 percentage points when compared against non-Hispanic White women. In 2017, 91% of the hospitals participating in the project were pursuing BFH designation and, by 2018, 14 hospitals had achieved their BFH designation [53]. Two articles examined the association between maternity care practices (MCP) and BF outcomes by race/ethnicity using PRAMS survey data [54, 55]. A retrospective observational study by Ahluwalia et al. [54] analyzed 2004–2006 PRAMS survey data from 11 states and NY City to evaluate the effect of MCP on BF duration (≥10 weeks vs. < 10 weeks). Different MCPs were associated with BF duration among racial/ethnic groups. BF within the first hour postpartum was associated with BF duration of ≥10 weeks among Black (OR 1.62; 95% CI 1.20–2.19) and non-Hispanic White women (1.36; 1.16–1.58). Likewise, BF on demand was significantly associated with BF duration of ≥10 weeks among Black (1.72; 1.28--2.31) and Hispanic women (1.49; 1.15–1.91). Across all racial groups, the number of newborns receiving only breastmilk at the hospital was associated with BF for ≥10 weeks. Three MCPs were also associated with less likelihood of BF for ≥10 weeks among Black women including: giving pacifier at the hospital (0.58; 0.44–0.77), receiving BF information (0.62; 0.34–1.12), and receiving help from hospital staff with BF (0.63; 0.46–0.88). Among Hispanic women, receiving a gift pack containing infant formula was associated with less likelihood of BF ≥ 10 weeks (0.67; 0.50–0.90). Among non-Hispanic White women, giving pacifier at the hospital was identified as a risk factor for longer BF duration (0.76; 0.66–0.87). The authors concluded that in order to improve BF outcomes among all women there is a need to better assess and understand the impact of MCP on women from different racial and ethnic backgrounds [54]. Another observational study by Sebastian et al. [55] used PRAMS survey data from New Mexico (2012–2014) and found that, compared with White women, Native American and Hispanic women were less likely to report BF while at the hospital and report receiving a phone number for BF support. Across all racial groups, offering only breastmilk at the hospital and BF on demand were positively associated with BF at 2 months. Hispanic women had the lowest BF rates compared with non-Hispanic White or Native American women. However, Spanish-speaking Hispanic women had similar BF rates at 2 months as non-Hispanic White women. Thus, findings suggest that BF services at hospitals should account for acculturation level among the Hispanic women that they serve.

Four studies supported the importance of BFH practices on BF outcomes. Nobari et al. [56] used 2012–1014 LA County triennial WIC random survey data and found that children born in BFH, compared to those born in non-BFH, were: (a) more likely to be EBF at 1 and 3 months, (b) more likely to have been breastfed only at the hospital, and (c) less likely to have been given formula at discharge. Consistent with these findings, another retrospective study by Jung et al. [57] using 2008–2017 data from same survey found that the uptake of BFH practices coincided with significant increases in any BF and EBF outcomes among WIC infants in Los Angeles County. A third study, an observational prospective cohort study conducted by Linares et al. [58] with Hispanic immigrant women at a Kentucky Hospital, found that skin-to-skin contact (SSC) at birth was associated with higher rates of EBF at discharge compared with those that did not have SSC (86.3% vs. 13.7%, *p* < 0.001), and this was independent of the type of delivery. Lastly, an observational prospective study by Ekambaram et al. [59] assessed the effect of implementation of a pacifier limiting in-hospital policy on BF outcomes. The study, conducted with predominantly African American women, found that the policy of limiting pacifier use at the birthing hospital was associated with higher EBF rates at hospital discharge, but not with BF rates at one month postpartum. However, this study must be interpreted with caution as, during the time that the pacifier policy was implemented, additional BFH training for all maternity hospital health also occurred.

Overall, these studies suggest that MCP based on The Ten Steps, were positively associated with BF outcomes, and positively impacted minority populations by decreasing disparities. They also suggest that the influence of these practices on BF outcomes may be modified by race/ethnicity, calling for BF services to account for the needs and wants of different racial/ethnic groups. These seven studies were of low (*n* = 6) or very low (*n* = 1) quality (Table 4).

**Table 4:** Community level healthcare facility breastfeeding interventions among women of color in the U.S.: Maternity ward

Study/Setting	Study Design/ methods	Study Population	Exposure/Intervention	Outcomes and Results	Grade
Merewood et al^.^ [53] Maternity Hospitals	**Design:** Prospective *N* = 33 hospitals **Data:** 2014–17 hospital data	4 Southern states; *N* = 31 hospitals; *N* = 210–3953 records; 2–85% AA; 0–90% H, 5–81% W	at least 3 BFH steps	BF initiation and EBF increased. Inequities in BF initiation between AA and W decreased by 9.6% points 14 hospitals received BFH designation.	Low
Ahluwalia et al. [54] PRAMS survey	**Design:** Retrospective **Data:** 2004–2006 Pregnancy Risk Assessment Monitoring System (PRAMS) survey	*N* = 49,135 women in 11 states and NY City 56.5% W; 14.4% AA, 23.4% H	Maternity Care Practices (MCPs)^a^	BF ≥ 10 weeks associated with: early BF initiation (AA & W); BF on demand (AA & H); only breastmilk in hospital (all). BF < 10 associated with: in-hospital pacifier (AA & W), BF support from hospital staff (AA); gift formula pack (H)	Low
Sebastian et al. [55] PRAMS survey	**Design:** Retrospective **Data:** 2012–2014 New Mexico PRAMS survey	*N* = 4035 Women who initiated BF; 8.4% Spanish-speaking H; 46% English-speaking H; 13.3% AI; 28.2% W	Supportive vs unsupportive BF MCPs	AI and H less likely to report BF at the hospital and receiving phone number for BF support. English-speaking H least likely to BF, only breast milk, staying in the same room, more likely to receive a gift formula pack.	Low
Nobari et al. [56] WIC survey	**Design:** Observational **Data:** Los Angeles County (LAC) WIC Survey (2008, 2011, 2014)	*N* = 1661 children ≤2 y old born at LAC hospitals (BFH; BFH in process; not BFH); 82% H	Exposure to any of 3 MCPs: Only breastmilk at hospital; Hospital gave gift formula; provided phone number for BF support.	Designated and in-process BFH associated with EBF at 1 and 3 mo.	Low
Jung et al. [57] WIC survey	**Design:** pre/post Observational **Data:** WIC triennial survey (2008, 2011, 2014, 2017)	*N* = 6449 eligible clients. 74% H	Exposure to any of 3 MCPs: Only breastmilk at hospital; Hospital gave gift formula; provided phone number for BF support.	Children born in a BFH (designated or in process) significantly higher in 2017 vs. 2014. Less formula packs in 2017. Any BF & EBF at 1 mo higher in 2017. BF at 6 mo and EBF at 1, 3, and 6 mo associated with only BF at hospital.	Low
Linares et al. [58] Medical chart	**Design:** Prospective **Data:** Pregnancy survey, medical charts, 1 mo p.p.	*N* = 97 H; Central Kentucky	Skin-to-Skin Contact (SSC) SSC vs. non-SCC	Higher EBF at discharge and at 1-mo p.p.; not associated with EBF 1-mo p.p.	Low
Ekambaram et al^.^ [59] Medical chart	**Design**: Pre/post observational **Data:** medical charts, 1 mo p.p. phone survey	*N* = 283 pre; *N* = 272 post; Philadelphia, Pennsylvania; 100% AA	Pacifier policy: not offering pacifier at the clinics and BF education.	Intervention associated with EBF on day of discharge; no differences on EBF at 1 mo p.p.	Very Low

^a^1.Hospital staff gave me BF information 2. My baby stayed in the same room with me at the hospital, 3. I breastfed my baby in the hospital (not included in analysis), 4. I breastfed my baby in the first hour after my baby was born, 5.Hospital staff helped me learn how to breastfeed, 6. My baby was fed only breast milk at the hospital, 7. Hospital staff told me to breastfeed whenever my baby wanted, 8. The hospital gave me a gift pack with formula, 9. The hospital gave me a telephone number to call for help with BF, 10. My baby used a pacifier in the hospital. Abbreviations: *AA* African American; *H* Hispanic; *AI* American Indian; *W* White; *BFH* Baby Friendly hospital; *MCPs* maternity care practices; *p.p.* postpartum; *EBF* exclusive breastfeeding; *BF* breastfeeding

#### Prenatal and post-partum interventions

Messito et al. [60] conducted a randomized controlled study with Hispanic women to evaluate the impact of the Starting Early Program (StEP), which focused on building mother’s responsive infant feeding behaviors skills and knowledge through a combination of group sessions and individual counseling during pregnancy and post-partum. StEP was associated with an 50% increase in continued BF at 10 months. A randomized intervention by Kao et al. [61] sought to prevent post-partum depression among a diverse population of low-income women. Within the intervention, nurses taught pregnant women the importance of self-care and being assertive (e.g. asking for help and support to be able to sustain BF) and offered a support post-partum session. While no differences were found between intervention and standard care groups regarding BF initiation, intervention mothers had a significantly longer BF duration compared to standard care mothers (54 vs. 21 days, respectively *p* = 0.013).

Watt et al. [62] conduced a quasi-experimental intervention among low-income Hispanic women that provided prenatal and post-partum nutrition education plus cooking demonstration, lactation counseling classes, and farmer’s market vouchers. Findings showed that the intervention improved BF rates compared to the comparison group (79.2% vs. 54.2%, respectively). Yet, this difference was not statistically significant (*p* = 0.66) which was likely due to the small sample size (only about 30% of participants participated in the post-partum component) and that the intervention focused more on improving overall family nutrition than just BF. A separate quasi-experimental pre/post implementation study conducted by Schreck et al. [63] assessed the influence of prenatal BF education and hospital-based BF post-partum support groups among a predominantly African American women. Improvements in BF initiation rates (71.9% post-intervention versus 51.5% at baseline (*p* < 0.0001)) were seen, but no associations were found with BF duration. Lastly, Efrat et al. [64] conducted a randomized study among Hispanic women during pregnancy and post-partum. A phone-based intervention was delivered by lactation educators who were undergraduate students with no BF experience. The intervention did not impact BF outcomes although the authors concluded that their intervention was a cost-effective strategy to in-person peer counselors (PCs).

Findings from two of the studies reviewed in this section [60, 61] suggest that the prenatal and postpartum interventions that were most effective at improving BF outcomes were those that taught skills to mother to improve responsive infant feeding or maternal self-care behaviors even though in both studies these BF was a secondary outcome. The 5 studies reviewed in this section were of moderate (*n* = 1), low (*n* = 2) or very low (n = 2) quality (Table 5).

**Table 5:** Community level healthcare facility breastfeeding interventions among women of color in the U.S.: Prenatal and post-partum

Study/Setting	Study Design/Data collection	Study Population	Intervention	Outcomes Results	Grade
Messito et al. [60] Pediatric clinics	**Design:** RCT **Data:** 3rd trimester baseline, chart review and 10 mo p.p. phone survey	*N* = 533 H (266 intervention and 267 controls), NY	1 prenatal & 8 p.p. individual and group nutrition counseling; delivered by bilingual RD/CLC	Intervention group were more likely to only use breastmilk as milk source for their infant at 10 months p.p.; compared to mothers in the control group	Moderate
Kao et al. [61] Prenatal clinic	**Design:** RCT **Data:** Baseline, post group sessions, 2 wks p.p. and 3 mo p.p.	*N* = 99 (53 interv. and 46 control); 44.4% H, 28% W, 14.1 AA; inner-city hospital in the Northeast	Intervention to prevent p.p. depression and increase social support, 4 group and 1 individual p.p.session	No between-group differences for BF initiation. Intervention group BF for longer than controls (54 days vs. 21 days). Intervention group twice as likely to still BF at 3 mo p.p.	Very Low
Watt et al^.^ [62] Primary Care clinic	**Design:** Quasi-experimental prospective **Data:** Surveys at 1st and 3rd pregnancy trimester; 2, 6, & 12 mo p.p.	*N* = 61 H (32 interv. and 20 comparison group); Southwest	Nutrition and cooking group classes in pregnancy and at 1 and 3 mo p.p.	At 6 mo mothers from intervention group marginally more likely to BF	Low
Schreck et al. [63] Prenatal clinic	**Design:** Pre/post quasi-experimental study **Data:** medical record review, phone survey at 1, 3 mo p.p.	*N* = 330 pre; 84.5% AA, 9.5% W *N* = 320 p.p.; 89.7% AA, 9.5% W	10 prenatal BF education sessions by an IBCLCs and 1 p.p. BF support group	BF education positively associated with BF initiation. Intervention mothers more likely to BF and achieve prenatal BF goals.	Low
Efrat et al. [64] Community Health clinics with WIC programs	**Design:** RCT **Data:** phone surveys at 3rd trimester, 72 h p.p., 1, 3 and 6 mo.	Intervention *N* = 111 H; Control *N* = 109 H; LA County	4 phone calls in pregnancy and 17 p.p. calls from lactation educator providing BF education and support up to 6 mo p.p.	No differences in BF initiation, EBF, any BF at 72 h, 1,3, 6 mo.	Very low

Abbreviations: *AA* African American; *AI* American Indian; *H* Hispanic; *W* White; *p.p.* postpartum; *RCT* Randomized Controlled Trial; *EBF* exclusive breastfeeding; *BF* breastfeeding; *RD* Registered Dietitian; *CLC* Certified Lactation Counselors; *IBLC* International Board Certified Lactation Consultant

#### Prenatal, hospital and post-partum interventions

Six randomized trials conducted during prenatal, hospital, and the postpartum periods found mixed results. Wambach et al. [65] assessed the impact of an intervention with low-income, primiparous predominantly African American adolescents (15–18 years old) delivered by a team of a nurse (who also was a lactation consultant) and a peer educator. This intervention lasted from second trimester of pregnancy until 4th week postpartum and had a positive impact on BF duration but not on BF initiation or EBF. Edwards et al. [66] conducted a trial also with young African American women and found that a doula program providing services such as prenatal home visits, BF support at birth, and post-partum support for 3 months with home visits and phone calls was associated with more BF attempts at the hospital as well as BF duration for more than 6 weeks but not BF duration at 4 months. Petrova et al. [67] conducted a study at a maternal and pediatric clinic in New Jersey with Hispanic women and found that prenatal education at the clinic plus additional in-hospital and postpartum BF support by phone from a lactation consultant was not associated with increases in EBF. In contrast to the previous study, Gross et al. [68] conducted a study with pregnant Hispanic women that resulted in the intervention being positively associated with EBF at 3 months (OR 1.61; 95%CI 1.07–2.44) [68]. In this case, this intervention consisted of BF education/counseling and support delivered by a team of bilingual registered dietitians (who were also Certified Lactation Counselors) during the third trimester of pregnancy, the hospital stay, and post-partum during child well visits. Similarly, Linares et al. [69] found that a culturally tailored intervention for Hispanic women, that included prenatal education, hospital BF support with home visits, and follow-up phone counseling until 6 months postpartum delivered by a PC and supported by a lactation consultant, increased EBF. This study found that participants in the intervention group were three times more likely to EBF their baby at discharge, 1 month, 3 months, and 6 months postpartum. However, the study had a 30% attrition rate at 6 months and a small initial sample. Lastly, Chapman et al. [70] provided BF specialized peer counselling support services for low-income overweight/obese women (predominately of Hispanic origin) delivering at a BFH, and did not find improvements in EBF or BF continuation at 1, 3, or 6 months postpartum among women who received the intervention. Nonetheless, adjusted post-hoc analyses showed that participants in the intervention group at 2 weeks postpartum had a significantly increased probability of continuing any BF (OR: 3.76 95% CI: 1.07–13.22), and offering 50% of feedings as breast milk (OR: 4.47 (95% CI: 1.38–14.5) compared with controls (who received BF PC non-specialized support).

The studies reviewed in this section suggest that interventions delivered in combination with PCs and other healthcare providers at multiple points of contact (prenatal, maternity hospital and postpartum), can have a positive impact on BF outcomes. Studies were of moderate (*n* = 2), low (*n* = 3) or very low (*n* = 1) quality (Table 6).

**Table 6:** Community level healthcare facility breastfeeding interventions among women of color in the U.S.: Prenatal, in-hospital and post-partum

Study/Setting	Study Design/ methods	Study Population	Intervention	Outcomes Results	Grade
Wambach et al. [65] Prenatal clinics	**Design:** RCT **Data:** 2nd trimester; 3, 6 wks & 2–6 mo p.p.	n = 128 intervention *n* = 128 control 1 *n* = 134 usual care; AA adolescents	IBLC and PC provided prenatal, hospital, and p.p. BF support, through 4 wks p.p.	Intervention had a positive effect on BF duration but not on BF initiation or EBF duration	Moderate
Edwards et al. [66] Prenatal clinic	**Design:** RCT **Data:** pregnancy baseline, hospital and 4 mo p.p.	n = 124 intervention *n* = 124 control group; AA	Doula conducted BF prenatal home visits, birth, 3 mo p.p.	Doula services increased in-hospital BF attempts and BF > 6 weeks but not at 4 months among young AA	Low
Petrova et al. [67] Health Center	**Design:** RCT **Data:** 3rd trimester, 7 days, 1,2,3 mo p.p.	n = 52 intervention *n* = 52 control; 87.5% H; New Brunswick, NJ	Prenatal BF classes, in hospital and p.p. BF support from IBLC by phone	No differences in BF initiation or EBF at 7 d., 1,2,3, mo p.p.	Low
Gross et al^.^ [68] Primary care Prenatal/Pediatric Clinic	**Design:** RCT **Data:** 3rd trimester, chart review, 3 mo p.p.; phone survey	n = 266 intervention *n* = 266 control; 100% H	RD/LC delivered prenatal education and provided p.p. BF counseling	Intervention increase EBF prevalence and BF intensity after discharge but not during the hospital stay.	Moderate
Linares et al. [69] Primary Health Care clinic	**Design:** RCT **Data:** 2 prenatal surveys, chart review; 1, 3 mo p.p. survey	*n* = 20 intervention *n* = 19 control; 100% H	BF prenatal education by IBLC & PC with home visits, BF support at Hospital,1st and 2nd wk. p.p.	Intervention increased EBF at all points. No differences in BF initiation.	Very low
Chapman et al. [70] Prenatal Clinic	**Design:** RCT **Data:** baseline survey, 3rd trimester phone survey, chart review, 24 h, survey at 2 wks and monthly thereafter until 6 mo p.p.	*n* = 76 intervention; 80% H women *n* = 79 Control; 83.3% H; overweight women; Hartford, CT	IBCL & PC prenatal BF education, in-hospital BF support, 11 p.p. PC home visits; supported BF among obese.	Intervention impacted any BF and BF intensity at 2 weeks p.p. but not with EBF Intervention infants less likely to be hospitalized after discharge	Low

Abbreviations: *AA* African American; *AI* American Indian; *H* Hispanic; *W* White; p.p. postpartum; *RCT* Randomized Controlled Trial; *RD* Registered Dietician; *LC* Lactation counselor; *IBCLC* International Board Certified Lactation Consultant; *PC* peer counselor; *EBF* exclusive breastfeeding; *BF* breastfeeding

#### Post-partum interventions

Six randomized trials included only post-partum interventions. The first was conducted by Pugh et al. [71] with low-income African American women recruited at a birthing hospital. The intervention, consisted of a BF support team formed by a community nurse and a PC providing intensive BF support during the first 4 weeks postpartum, and continued support until 24 weeks postpartum. The intervention was positively associated with BF at 6 weeks (OR 1.71; 95% CI 1.07, 2.76) but not 12 and 24 weeks. The second randomized study, conducted by Moon et al. [72] also with African American mothers, found that receiving information post-partum about bedsharing and risk of Sudden Infant Death Syndrome (SID) was not associated with BF or EBF rates at 2–3 weeks, 2–3 months or 5–6 months postpartum.

A third randomized study by Hopkinson et al. [73] included Hispanic immigrant women who were mix feeding in the hospital after delivery. The intervention, designed to increase EBF after discharge, found that at four weeks mothers from the intervention group were more likely to be EBF (OR = 1.87; 95% CI, 1.07–3.26). However, this change from mixed feeding to EBF was mainly due to the removal of pre-lacteals like tea or water and not a decrease in formula feeding. A fourth randomized study by Bunik et al. [74] conducted among low-income predominantly Hispanic women found that bilingual nurses providing daily telephone-based BF support (screening for lactation or medical problems) during the first 2 weeks post-partum was not associated with BF duration or exclusivity. The fifth study was a pragmatic trial conducted by Harris-Luna & Badr [75] with Hispanic post-partum women planning to breastfeed their babies. Promotoras administered BF telephone-based support during the first 12 weeks postpartum, and results showed the probability of EBF at 12 weeks increased more than threefold (3.39; 1.01, 11.46, *p* = 0.04). However, findings from this study need to be interpreted with caution since randomization procedures were not adequate as participants were assigned to intervention or control group sequentially based on their appointment time. The sixth randomized study was conducted by Howell et al. [76] with Hispanic and Black mothers receiving a behavioral intervention preparing them for the post-partum period by providing education about BF management, the importance of social support, and self-care during this sensitive period of time. The intervention had a positive impact on BF duration (median BF duration: 12.0 weeks in intervention vs. 6.5 weeks in control; *p* = 0.02).

Two retrospective studies yielded important findings. An observational retrospective study by Bream et al. [77] conducted with predominantly African American women found no association between reported use or request of a breast pump at infant’s first newborn visit (14 days postpartum) and EBF at 1.5–3.5 months. However, breast pumps were associated with increased use of expressed breast milk and decreased feeding at the breast. Thus, using breast pumps was associated with lower rates of any BF at 1.5–3.5 months. In a retrospective multi race/ethnicity study, Mercier et al. [78] documented that Medicaid-insured mothers were significantly less likely to EBF or provide any BF at 6 to 8 weeks postpartum compared with commercially insured mothers.

The studies reviewed in this section suggest on the one hand that BF interventions delivered during the postpartum period have mixed results. On the other hand, BF interventions delivered during the perinatal period that may have some positive effect on BF outcomes. Breast pumps may not increase BF but rather increase the proportion of feeds involving expressed breast milk. Studies were of moderate (*n* = 2), low (n = 2) or very low (*n* = 3) quality (Table 7). Furthermore, the study by Howell, et al. [76], illustrates the importance of including appropriated behavior change frameworks when designing and evaluating BF interventions targeting minority women [79].

**Table 7:** Community level healthcare facility breastfeeding interventions among women of color in the U.S.: Post-partum

Study/Setting	Study Design/ methods	Study Population	Exposure/Intervention	Outcomes Results	Grade
Pugh et al. [71] Urban hospitals	**Design:** RCT **Data:** baseline, 6, 12, 24 wks p.p. phone/home survey	*n* = 168 intervention *n* = 160 control 87% AA	Three 1-4th wks p.p. PC home visits; PC bi-weekly phone calls, nurse available 24/7 by pager first 24 wks p.p.	At 6 wks p.p., intervention mothers 1.71 times more likely to BF. No significant %BF differences at 12 and 24 wks p.p.	Low
Moon et al. [72] Urban Hospital	**Design:** RCT **Data**: surveys; 1–2 d p.p., 2 wks, 2–3 mo, 5–6 mo p.p.	*n* = 569 intervention *n* = 625 control 100% AA	Information on AAP safe sleep practices to reduce SIDS risk	No impact on BF or EBF rates despite emphasis on not bedsharing.	Very Low
Hopkinson et al. [73] Community clinic	**Design:** RCT **Data:** Chart review, baseline survey, phone at 4 wks p.p.	*n* = 255 intervention *n* = 267 control. 87.5% H	Moms mix feeding scheduled for a 3–7 p.p. visit to clinic; PC & IBCLC BF support;	Intervention increased EBF at 4 wks, mostly due to less water and herbal teas (vs. formula)	Moderate
Bunik et al. [74] Community health center	**Design:** RCT **Data:** Hospital, 1,3,6 mo p.p. phone survey	*n* = 161; 87% H *n* = 180; 90% H	daily BF support phone calls; bilingual nurses; 1st 2 wks p.p.	No differences in any or predominantly BF at 1, 3, 6 mo p.p.	Very Low
Harris-Luna & Badr [75] Obstetric clinics	**Design:** Pragmatic RCT **Data:** in-person baseline survey; 12 wks phone survey	n = 31 intervention *n* = 30 control; 100% H; Southern CA	Promotoras BF phone daily support 4 wks p.p., then biweekly until 12 wks p.p.	Increased EBF at 12 wks p.p.; longer BF duration	Very Low
Howell et al. [76] Tertiary hospital	**Design:** RCT **Data:** Baseline, 3 wks, 3, 6 mo p.p.	n = 270 intervention 64% H; 36% AA *n* = 270 Control 61% H; 39% AA; NY	p.p. behavioral education; BF tips, social support/self-management skills.	Longer BF duration; less likely to stop BF during first 6 mo p.p.	Moderate
Bream et al. [77] Pediatric clinic	**Design:** Retrospective **Data:** chart review first 14 d p.p., 2 mo p.p. survey	*N* = 355; 96% AA; Cleveland OH	BF mothers requesting a breast pump or using one at 14 days p.p.	Breast-pumps did not increase EBF at 2 mo; less BF at 2 mo p.p.	Very Low
Mercier et al. [78] University Hospital	**Design:** Observational Retrospective study **Data:** chart review; hospital & 6–8 wks p.p.	*N* = 405; 54% AA; 11% As; 11% H; Philadelphia	Exposure: Insurance status; Medicaid or commercial insurance	Among AA, no significant differences in BF rates AA with or without commercial insurance.	Low

Abbreviations: *A* Asian; *AA* African American; *AI* American Indian; *H* Hispanic; *W* White; *p.p.* postpartum; *PC* peer counselor; *RCT* Randomized Controlled Trial. *IBCL* International Board Certified Lactation Consultant; *PC* peer counselor; *EBF* exclusive breastfeeding; *BF* breastfeeding; *SIDS* Sudden Infant Death Syndrome

### The Special Supplemental Nutrition Program for Women, Infants, and Children (WIC) interventions

Marshall et al. [80] conducted a cross-sectional analysis of Mississippi’s 2004–2008 PRAMS data and found that WIC participation was negatively associated with BF initiation among non-Hispanic White women (OR 0.87; 95% CI 0.77–0.99), but not among Black women (OR 0.99; 0.28–1.21) [80]. In a retrospective observational study using Maryland’s 2007 WIC program administrative data (*n* = 33,582), Gross et al. [81] found that, at the time of postpartum WIC certification, meaning that the mother and child met the program’s eligibility low-income and other criteria to receive program benefits according to the status of the mother (pregnant versus postpartum) and infant feeding choices made by the mother; i.e. full BF, partial BF, formula feeding [82]. Hispanic infants were more likely to be certified as partially BF (49%) when compared with non-Hispanic White (11%) and African American infants (20.5%; *p* < 0.001). They were also more likely to be certified after 2 weeks of age (46.1%) when compared with non-Hispanic White infants (31.6%) and African American infants (28.6%; p < 0.001). At the time of certification, BF initiation rate was higher among women receiving peer counseling compared with those receiving a lactation consultant or standard of care services (61.6% vs. 54.4 and 47.6%, respectively; p < 0.001). In this study, peer counseling was also positively associated with being certified as exclusively and partially BF compared with the lactation consultants and standard of care groups (36.0% vs. 24.8 and 25.3%, respectively; p < 0.001).

There were four studies conducted among WIC participants where the intervention was compared with a group already receiving some WIC BF support services. The first one, conducted in Philadelphia by Washio et al. [83] with Puerto Rican mothers, provided monthly cash incentives to BF mothers to incentivize continuation. The incentives led to increased BF duration at 1 month (89% vs. 44%, *P* = 0.01); 3 months (89% vs. 17%, *P* < 0.001); and 6 months (72% vs. 0%, P < 0.001) post-partum. However, the intervention had no impact on EBF, possibly due to the low sample size for this analysis. The second and third studies focused on the same two-way text messaging BF counseling intervention, LATCH (Lactation Advice Through Texting Can Help). The LATCH pilot RCT, conducted by Harari et al. [84], was followed by a larger scale RCT conducted by Martinez-Brockman et al. [85]. Both studies were conducted in Connecticut through WIC clinics and one community agency. LATCH delivered automated and personalized BF behavior change text messages from pregnancy to post-partum period as a tool to enhance BF PC services. Both studies confirmed the feasibility of LATCH and underlined how helpful the intervention was for establishing contact with women significantly sooner after birth. Yet, no significant effect of this intervention was seen on EBF or any BF at 2 or 3 months postpartum. The fourth study was a quasi-experimental study conducted by Lovera et al. [86] in Texas. Fathers of breastfed infants were hired as peer dads to provide BF information and support to other fathers with breastfed infants participating in WIC and receiving BF peer counseling services. The peer dad program was not associated with BF duration.

Three additional studies assessed different modalities to deliver or enhance WIC BF support services [87, 88]. In a randomized study in Oregon at 4 WIC offices (2 rural and 2 urban sites), Reeder et al. [87] assessed the impact of a telephone-based peer counselling service on BF outcomes as an alternative model to the more expensive in-person peer counselling model. Findings revealed that telephone calls from PC were associated with higher BF duration at 3 months postpartum (AOR 1.22; 95% CI: 1.10–1.34). However, at 6 months, this relationship was only found among Spanish-speaking clients (1.29; CI: 1.10–1.51). Edmunds et al. [88] conducted a quasi-experimental study where pregnant women intending to breastfeed their babies were recruited from 12 WIC clinics to participate in the *You Can Do It* intervention. In the intervention, women received a tailored individual plan with BF counseling from pregnancy through the immediate postpartum period. The intervention was positively associated with EBF at 7 days (OR 1.6; 85% CI 1.1–2.5) and 30 days postpartum (1.6; 1.0–2.5), but not at 60 days. When examined by race/ethnicity, the positive impacts on EBF rates were documented among Black and Hispanic at 30 and 60 days postpartum but not non-Hispanic White women. Another simple quasi-experimental intervention examined the effect of a bilingual video used at a WIC office in NY City that encouraged mothers to BF, however no impact on BF outcomes was seen [89].

The studies reviewed in this section suggest that, even though the WIC population is at high risk of poor BF outcomes, there is still a dearth of consistent evidence on effective BF interventions that can be delivered through WIC clinics. Overall, postpartum interventions delivered by PCs have a positive impact on BF outcomes. In this section we identified interventions designed to complement WIC BF support services already offered at WIC offices that included financial incentives and behavior change based text messaging. The latter interventions seem promising but it is important to continue exploring the use of information technologies, including telehealth, to strengthen the reach and depth of peer counseling and other BF support services at WIC. Studies were of moderate (*n* = 1), low (*n* = 5) or very low (*n* = 3) quality (Table 8).

**Table 8:** Community level Supplemental Nutrition Program for Women, Infants, and Children (WIC) breastfeeding interventions among women of color in the U.S.

Study/Setting	Study Design/ methods	Study Population	Exposure/Intervention	Outcomes/Results	Grade
Marshall et al. [80] PRAMS data	**Design:** Retrospective **Data:**2004,2006,2008 Mississippi PRAMS	*N* = 1739 W; 52.2% *N* = 1975 AA; 82% enrolled in WIC Mississippi	WIC vs. eligible non-WIC participants	WIC participation not associated with BF initiation among A	Low
Gross et al. [81] WIC	**Design:** Retrospective **Data**: WIC	N = 33,582; 50.5% AA; 23.8% H; 22.1% W; Maryland	BF support by PC, IBLC vs. standard of care (SC)	BF initiation rate higher for PC vs. IBLC and SC groups.	Very Low
Washio et al. [83] WIC clinic	**Design:** RCT **Data:** Baseline, 1,3,6 mo	n = 18 intervention; *n* = 18 control, 100% Puerto Rican; Philadelphia	WIC BF support services plus incremental financial incentives per month BF	Higher BF at 1, 3, and 6 mo vs. controls	Low
Harari et al. [84] WIC clinics	**Design:** pilot RCT **Data:** baseline intake; 2 wks p.p. phone survey	n = 32 intervention; n = 26 control; 75% H;17% AA; 6% W; New Haven CT	Personalized text messages + BF PC services during pregnancy and p.p.	EBF at 2 weeks higher in intervention (NS). Intervention mothers were contacted sooner and more likely to meet BF goals	Moderate
Martinez-Brockman et al. [85] WIC clinics	**Design:** RCT **Data:** baseline, 2 wks, 3 mo p.p. phone surveys	*N* = 94 intervention *n* = 80 control 74.3 H%; CT	PC services + two-way text messages from pregnancy to 3 mo p.p.	EBF rates at 2 wks p.p. higher (NS). Intervention moms contacted sooner. NS between-group differences in EBF at 3 mo	Low
Lovera et al. [86] WIC clinic	**Design:** Quasi experimental **Data:** WIC records	n = 53 intervention; 100% H; n = 49 control 100% H; Brownsville TX	PC + peer dad counseling services	NS between-group difference in BF duration	Very low
Reeder et al. [87] WIC clinics	**Design:** RCT **Data:** 1st trimester, 7,30,60 days p.p.	*n* = 645 PC high freq. *n* = 646 PC low freq. *n* = 657 control; 100% H; 4 clinics; Oregon	Low freq. PC group: 4 calls; High freq. PC group: 8 phone calls	Intervention increased EBF and any BF duration among Spanish speaking WIC participants. NS among English speaking.	Low
Edmunds et al. [88] WIC clinics	**Design:** Quasi-experimental. **Data:** 1st trimester, 7,30,60 days p.p.	*n* = 362 intervention; *n* = 408 control; 11.1% W, 30.7% AA, 51.2% H, 12 clinics; NY State	Individual counseling tailoring	Intervention increased EBF among H and AA at 30 & 60 days p.p.	Low
Scheinmann et al. [89] WIC clinics	**Design:** Quasi-experimental. **Data:** baseline, 3, 6 mo p.p.	*N* = 439; 100% H	25-min English/Spanish educational BF video	NS differences in any BF between video and comparison groups at baseline, 3 & 6 mo	Very low

Abbreviations: *AA* African American; *H* Hispanic; *W* White; *NS* not significant; *p.p.* postpartum; *PC* peer counselor; *RCT* Randomized Controlled Trial

### Community agency interventions

Four community intervention studies were randomized controlled trials. The first study was conducted by Cloutier et al. [90] in Hartford, CT with a predominantly Hispanic population receiving enhanced services with a BF component from the Nurturing Family Network (NFN). Despite the small sample and high attrition rate (28%) (half in the control group were lost to follow-up at 6 months), the intervention increased BF at 6 and 12 months (but not BF initiation) compared to those mothers not receiving the intervention. The second randomized study, conducted by Sandy et al. [91] in New York among low-income Hispanic immigrant women, showed that a BF education and support intervention offered during pregnancy and postpartum through a home visiting maternal program had a positive impact on EBF during the first week post-partum but did not have a significant impact on BF duration. However, EBF was measured based on only breastmilk with no formula without assessing pre-lacteal feedings. The third randomized study was conducted by Lutenbacher et al. [92] among Hispanic women and assessed the impact of a home visiting program utilizing peer educators from pregnancy through 6 months. The study found that the intervention group had a longer duration of EBF compared to the control group (1.4 vs 0.3 weeks, respectively). The fourth randomized study, conducted by Hans et al. [93] in Illinois, found a positive impact of a doula home visiting program on BF initiation (81% vs. 74%; OR = 1.67, 0.91–3.03, *p* = .05), but not on BF rates at 3 months (16.9%vs. 21.8%; OR = 0.85, 0.45–1.60) .

Four community intervention studies were quasi-experimental studies. Karanja et al. [94] designed an intervention to limit sugary drink intake and improve BF practices among AI/AN toddlers. Findings showed that a social media campaign combined with a home-based community health worker (CHW) intervention was associated with higher BF initiation and BF duration rates at 6 months when compared to national data for the AI/AN populations. Thomson et al. [95] tested an early childhood obesity prevention intervention focused on gestational weight gain, BF, mother and infant nutrition, and physical activity with clients of the Parents as Teachers (PAT) home visiting program, who were high school and college educated African American women living in several Mississippi counties. The intervention did not significantly impact any BF outcomes. In the Moms First Project with African American women, Furman et al. [96] tested a pregnancy and postpartum intervention that included doula services and a lactation counselor providing BF support through home visits and phone calls. Among intervention participants, any BF was associated with a higher receipt of curricular modules and number of post-partum home visit, while EBF was only associated with a higher number of post-partum home visits. Results from this study only included women receiving an intervention since there were incomplete BF outcome data from control participants. Lastly, Lewkowitz et al. [97] tested a prenatal home-based lifestyle intervention by parents to overweight/obese African American pregnant women using an enhanced (i.e. included a BF component) Parent as Teacher Curriculum. The intervention did not impact BF initiation rates.

Three community interventions were observational studies. First, a study conducted by Leruth et al. [98] among African American women, found that a Healthy Start Program (HSP) in Chicago, Illinois that incorporated BF education and support by certified lactation counselors (CLC) from pregnancy through 6 months postpartum had a positive impact on BF initiation. However, results must be interpreted with caution since the program lacked baseline BF data before the incorporation of BF supportive services. Instead, BF initiation rates from the study were compared to one partner hospital receiving the program services the year before the implementation of BF support services to the HSP. Second, in a study conducted with a diverse group of Medicaid recipient women who received doula services during the perinatal period, Kozhimannil et al. [99] reported a high BF initiation rate (97.9%) among those receiving this service when compared with state based data of Medicaid recipient women not receiving this service (80.8%). Furthermore, doula services had a significant impact on BF initiation rates among African American women which were 92.7% compared with 70.3% of the general Medicaid population not receiving the services. Lastly, a comparative observational study assessing the efficacy of implementing a perinatal home visiting program using mother-to-mother support (regarding BF and other postpartum topics) during the perinatal period did not impact BF outcomes [100].

Collectively, the studies reviewed in this section strongly suggest that community-based interventions delivered through community agencies and that include home visits can have a positive impact on BF outcomes among minority women. Almost all studies focused on African American or Hispanic women. Studies were of moderate (*n* = 1), low (*n* = 3) or very low (n = 3) quality (Table 9).

**Table 9:** Community level community agencies breastfeeding interventions among women of color in the U.S.

Study	Study Design	Study Population	Intervention	Outcomes	Grade
Cloutier, et al. [90] Home visiting program	**Design:** RCT **Data**: baseline, 6, 12 mo p.p. survey	n = 30 intervention: 24% AA; 52% H; 24% Other; n = 27 control: 24% AA; 52% H;24%; 6 sites; Hartford CT	Standard NFN curricula plus enhanced BF/Responsive parenting modules.	No impact on BF initiation; positive impact on BF at 6 and 12 mo	Very low
Sandy, et al. [91] Home visiting program	**Design**: RCT **Data:** baseline, 1 wk. p.p.	*n* = 137 intervention, *n* = 101 control; all H mostly Dominican; NY City	Prenatal home visits by FSWs; SC curricula + BF education + BF support, pediatric resident visit 1 wk. p.p.	EBF but not any BF associated with intervention at 1st week p.p.	Very Low
Lutenbacher et al. [92] Home visiting program	**Design**: RCT **Data:** baseline, 2 wk., 2 & 6 mo p.p.	n = 94 Intervention; *n* = 94 controls; all H; Tennessee	Maternal Infant Health home visiting Program vs. only written information	Intervention improved EBF duration	Moderate
Hans et al. [93] Community agencies	**Design**: RCT **Data:** 3rd pregnancy trimester, 3 wks & 3 mo p.p.	*N* = 129 Intervention: 43.6% AA, 39.1% H, 8.3% W; n = 27 control: 46.2% AA, 35.9% H, 8.3% W; *n* = 4 agencies	Home visitor + doula; pregnancy and up to 6 wks p.p.	Intervention improved BF initiation; no impact on BF at 3 months p.p.	Low
Karanja et al. [94] Home visiting program	**Design**: Quasi-exp. **Data**: national data^a^, 6 mo survey, visit logs, WIC records	*N* = 142 mother and child All AI/AN; Oregon	8 CHWs home visits; pregnancy thru child second y	BF initiation and 6-mo rates higher than national average	Low
Thomson et al. [95] Home visiting program	**Design**: Quasi-experimental. **Data**: 2nd pregnancy trimester, 12 mo p.p.	*n* = 39 intervention, *n* = 43 control 96.3% AA, Mississippi	PAT home visiting curriculum + culturally tailored pregnancy weight gain and early childhood obesity prevention lessons	No effect of intervention on BF outcomes	Very Low
Furman et al. [96] Home visiting program	**Design**: Quasi-exp **Data**: intake survey, home visits and phone calls logs	*n* = 1000 in interventions 84.3% AA; *n* = 296 no intervention; 80% AA; Cleveland	Home visiting program and/or Doula or father’s evening program	No effect of interventions on BF outcomes. More home-visits increased EBF and any BF odds.	Very low
Lewkowitz et al. [97] Home visiting program	**Design**: Quasi-experimental. **Data**: phone survey; 6–12 mo. p.p.	n = 59 intervention; *n* = 59 control; all AA; St. Louis Missouri	Parent educator home visits; parenting curricula + healthy lifestyle & BF education	No effect of intervention on BF outcomes	Low
Leruth et al. [98] Office/Home visiting program	**Design**: Pre/post **Data**: pre- BF program data; baseline and 6 mo p.p. post-BF data	*n* = 280; mostly AA Chicago, Illinois	Healthy Start BF model; home/office based services + BF counseling pregnancy to 6 months p.p. delivered by a CLC	Higher BF initiation and %any BF at 6 mo. after incorporating BF component.	Very low
Kozhimannil et al. [99]	**D.esign**: Observational **Data**: 2009/2010 PRAMS and Medicaid, program’s logs	*n* = 1069 intervention 10.1% W; 46.6% AA; 36.5% H; 5.5% Asian; 1.0%AI; 0.3% other. *n* = 51,721 PRAMS	Pregnant women with Medicaid coverage vs. pregnant women with Medicaid coverage + Doula from non-profit organization	Higher percentage of women who had doula-supported births-initiated BF	Low
Rotheram-Fuller et al. [100] Home visiting program	**Design**: RCT **Data**: surveys; baseline, 2 wks p.p., 6 mo p.p.	n = 99 intervention *n* = 104 controls; 80% H; LA	Home visiting BF Peer Mentor Mothers (MM)	No effect of intervention on BF outcomes	Very low

Abbreviations: *AA* African American; *AI* American Indian; *AN* Alaskan Native; *H* Hispanic; *W* White; *NS* not significant; *p.p.* postpartum; *PC* peer counselor; *RCT* Randomized Controlled Trial CHW, Community Health Worker; *NFN* Nurturing Family Network; *FSW* Family Support Worker; *PAT* Parent as Teacher; *CLC* Certified Lactation Counselor ^a^National Pediatric Nutrition Surveillance System (PedNSS) data for AI families

### Interpersonal level

Three studies (two cross-sectional and one experimental) were at the interpersonal level. First, in a cross-sectional study conducted with low-income, predominantly African American women, Ashida et al. [101] found that mothers that received more supportive rather than undermining BF advice were 1.8 times more likely to ever breast-feed (OR = 1.8; 95% CI 1.1–3·0). Furthermore, the presence of supportive BF information was significantly associated with a higher likelihood of ever BF (2·0; 1.0–3.8) [101]. Another cross-sectional study conducted by Thomas et al. [102] that interviewed women at a pediatric clinic found that mothers with an accompanying person were more likely to be exclusively BF when compared with those women without an accompanying adult (47% vs. 33%, respectively; *p* = 0.04). Finally, an experimental study conducted by Wilhelm et al. [103] did not find motivational interviewing to be effective at increasing self-efficacy and BF at 6 months among Mexican women living in rural areas. However, this study had a high attrition rate and small sample size [103].

These studies suggest that interpersonal support may improve BF outcomes among minority women and there is a need to conduct more research in this area. Studies were low (*n* = 1) or very low (*n* = 2) quality (Table 10).

**Table 10:** Interpersonal level breastfeeding interventions among women of color in the U.S.

Study/Settings	Study Design/methods	Study Population	Intervention	Outcomes/Results	Grade
Ashida et al. [101]	**Design:** Cross-sectional **Data**: 20-45minute survey	n=81 mothers; 80% AA; City in the South	Receiving supportive vs. undermining BF information from social networks	Participants who received more supportive than undermining BF advice were more likely to ever BF	Very Low
Thomas et al. [102]	**Design**: Cross-sectional **Data:** two 5-min surveys for mothers & accompanying person	n=192 mothers; 101 with accompanying adult	Mothers with vs. mothers without accompanying person	Mothers with accompanying person more likely to EBF	Low
Wilhelm et al. [103]	**Design**: RCT **Data:** 3 d, 2, 6 wks pp. surveys and 6 months phone interview.	n=26 intervention; n=27 controls; All H	BF motivational interviewing session at 3 d, 2 & 3 wks pp.	No between-group differences 3 BF at 6 mo	Very Low

Abbreviations: *AA* African-American; *H* Hispanic; *RCT* Randomized Controlled Trial; *pp.* postpartum

## Discussion

Over the past decade, BF rates have increased among all racial/ethnic groups in the U.S. but inequities still exist. Ethnic/racial minority women breastfeed exclusively for shorter periods of time [25] and are significantly less likely to meet their BF goals compared to non-Hispanic White women [104]. Our systematic review (SR) sheds some light as to why this may be the case following a socioecological framework.

### Policy level

Half of the 6 included articles included at the federal and state policy level assessed the impact of BF supporting laws on BF outcomes. Findings demonstrated that laws at the federal level influenced ever BF with the greatest benefit among minority women. Policies at the state level that required employers to provide adequate breaks and space benefited Hispanic by strengthening BF initiation and BF duration to 6 months plus improving ever BF among Black women. Furthermore, Black women were more likely to initiate BF in states where there were laws protecting BF in public and private locations [43]. However, unexpectedly, in a low quality observational study, Black women were less likely to continue BF for 6 months compared with White women in states with BF laws providing breaks, exemption for jury duty, and workplace pumping laws [44].

It is clear from these studies that racial/ethnic groups were affected differently by state-level BF supporting laws. This is not surprising because returning to work and low socio-economic status are both risk factors for suboptimal BF outcomes in the general population [105]. Indeed, in 2018, Black women in the U.S. were more likely to be working (62.4%) compared with White (57.6%%), Asian (58.6%), or Hispanic (59.4%) women. Among women with children under 18 years old, Black women (77.2%) were also more likely to be working than White (71.2%), Asian (65.0%), or Hispanic women (63.9%) [106] . Even though most BF workplace laws have limitations, including weak enforcing mechanisms, they were positively associated with BF initiation and with decreased inequities in this outcome. However, the effect sizes were small suggesting that maternity protection policies need to be strengthened to further support BF among minority women in the U.S. Since paid maternity leave has been associated with improved BF initiation and duration [107, 108], it is indeed important to develop strong, enforceable federal paid maternity leave policies in the U.S. These policies are needed by low-income women, including those belonging to ethnic/racial minority groups, many of which have jobs incompatible with BF. It is important to pay special attention to structural inequities in the types of employments related to immigration status. For instance, undocumented immigrants may have less access to any kind of maternity protection benefits as a result of informal employment.

Our findings also suggest that laws protecting women’s rights to breastfeed in public and private places facilitate BF among minority women. More research is needed to understand how to translate BF supportive policies into programs that are tailored to the needs of women following a socioecological model approach.

Changes to the Supplemental Nutrition Program for Women, Infants and Children (WIC) packages in 2009 designed to incentivize BF represent an important policy “experiment” that is highly relevant to ethnic/racial minority women. WIC is a food assistance federal program serving low-income pregnant and post-partum women, and their children under 5 years old. WIC serves over half of the annual births in the country and has strong representation from ethnic/racial minority groups. WIC program staff are required to provide nutrition education and anticipatory guidance on BF to pregnant women plus post-partum BF support to mothers who choose to breastfeed. WIC also offers food packages designed to supplement the nutritional needs of mothers and infants with healthy foods, and the 2009 package was also designed to incentivize BF. In our review, we found that the 2009 WIC package revisions seem to have a positive impact on BF practices. Specifically, an NHANES analysis found that the changes in the WIC package made WIC participant children to initiate breastfeeding at rates similar to non-WIC participating eligible children, while this was not the case for BF duration at 6 months [45]. Another pre/post study conducted in Los Angeles county among WIC participants, found that 2009 WIC package changes positively influenced BF initiation and the likelihood of EBF at 3 and 6 months [46]. A previous review found mixed results on the relationship between WIC 2009 food package revisions and BF outcomes in the general WIC population, and it is possible that these relationships are different across different states [109] . Hence, more research is needed to further elucidate if and how these changes affected BF outcomes across different states and ethnic/racial minority groups.

In summary, with regards to policy level interventions, we found that federal and state laws and policies supporting BF have indeed been positively associated with BF initiation among minority and low-income women. However, it is necessary to go beyond these policies to improve success with BF duration and EBF across minority and low-socioeconomic groups by considering paid maternity leave and access to high quality BF support at the health facility and community levels. The WIC program could play a key role by implementing additional package changes recommended by the National Academy of Medicine in 2016 to further incentivize BF and building from lessons learned from the 2006 WIC package revisions [110].

### Community level

At the community level of the socioecological model, we identified studies examining interventions or exposures driven by health care facilities, WIC, and community agencies.

#### Healthcare facilities

The majority of the BF interventions reviewed in this section of the SR were related to MCP based on the Ten Steps from the Baby Friendly Hospital Initiative (BFHI). Our review shows that these practices influence BF outcomes. The CHAMPS intervention, located in hospitals across 4 Southern states, found that the process of becoming a Baby Friendly Hospital was effective at improving BF outcomes in an ethnic/racially diverse group of women and closed the gap in BF initiation among African American women [53].

Feeding only breastmilk in hospital was strongly associated with longer BF duration among women from multiple ethnicities/races, but studies also documented differential responses as a function of maternal ethnicity/race. Unexpectedly, one study found that receiving BF information and support at the hospital was negatively associated with BF duration among Black women [54]. Another study found that Hispanic and Native American women were less likely to have given a telephone number to call if they needed BF support after hospital discharge [55] perhaps as a result of stereotyping of BF behaviors [20].

In addition, the provision of formula at the hospital and formula gifts at discharge was associated with suboptimal BF practices among Hispanic women [54]. This finding is consistent with previous studies that have found that Hispanic women tend to have a strong preference for mixed feeding [69, 111].

In our SR, we also found evidence that multi-point maternity care interventions that have prenatal, perinatal, and post-partum components including PCs together with International Board Certified Lactation Consultants (IBCLCs) or other health professionals can have a strong impact on EBF and/or any BF duration [65, 66, 69, 70] This agrees with previous literature reviews focusing on the global BF counseling evidence, including from the U. S. [35, 112, 113]

In summary, on the one hand, our SR indicates that maternity care practices consistent with the Ten Steps improve BF outcomes among minority women and can contribute to decreasing BF disparities. On the other hand, it documents the need to include cross-cultural training for maternity care staff to learn how to better tailor their support to the specific needs of Black and Hispanic women to improve BF rates, which is consistent with previous recommendations [35].

#### WIC

Participation in the WIC program has been consistently associated with lower BF rates when comparing WIC mothers with WIC-eligible women not enrolled in the program [114]. An analysis of 2004–2008 Mississippi PRAMS found that WIC participation was not associated with BF initiation or duration among Black mothers when compared with their White counterparts [80]. In our review, several RCTs tested the impact of enhancements to BF support services offered by on BF outcomes. An innovative study showed that increasing monthly financial incentives for BF among Puerto Rican women had a sizable and statistically significant positive impact on increasing BF duration but had no impact on EBF [83]. This is consistent with behavioral economics interventions that have focused on other nutrition behaviors including fruit and vegetable intake among low-income populations in the U.S. [115].

This review also included WIC interventions that focused on the use of information technology to support BF. A RCT found that two-way behavior change text messages initiated by PCs serving WIC mothers in Connecticut did not have a significant impact on EBF at 2 weeks post-partum perhaps due to sample size limitations; yet, the EBF rate was much higher in the intervention than in the control group. This intervention did facilitate establishing earlier contact with the mothers after birth, which is considered to be key for BF success as the onset of many of lactation problems happen during the first few days after birth [85]. Indeed, one of the studies that reviewed identified gaps in WIC BF support services found that by the time of post-partum WIC certification many mothers that had initiated BF were already offering mix feeding to their infants or had stopped BF altogether because they did not receive timely BF support. This observational study found that mothers that received peer counseling services were more likely to continue to breastfeed at WIC certification when compared with the other WIC BF support services [81].

Findings from a study on WIC conducted in NY state offices suggests that tailoring interventions to women’s individual needs is important for improving EBF rates among Hispanic and Black WIC participants. This is consistent with findings showing that women of different race/ethnicities respond differently to similar BF interventions [116].

#### Community agencies

Several BF interventions were delivered to diverse racial/ethnic groups during pregnancy and the post-partum period by community agencies through community health workers as part of their maternal and child home visiting programs. Overall, our findings indicate these programs were positively associated with BF duration among minority women. Because most of these studies were graded as having very low-quality, it is important that better designed studies are carried out to try to confirm these findings.

#### Interpersonal level

There was a dearth of intervention studies that examined the impact of BF support for minority women through their social networks. The few identified in our SR suggest that minority women’s support networks can influence the type of BF advice given by friends and relatives and, ultimately, BF practices. Although this is consistent with theoretical expectations [117], this is an area that needs to be better studied among minority women using mixed-method approaches.

Very few studies reviewed reported specific behavioral change strategies with psychosocial components, however, findings seem to suggest that behavior change driven interventions may have positive effects on BF duration among minority women. More studies among minority women are strongly needed in this area as they are key for learning how best to tailor BF interventions to the needs and wants of different ethnic/racial groups.

#### Research recommendations

Only 36% (*n* = 22) of the articles included in this review included women from more than one racial/ethnic group. Furthermore, 40% (*n* = 24) of the studies focused on Hispanic mothers only, the majority of which only included women of Mexican origin. About 22% (*n* = 13) of the studies were conducted among Black women and only 1.6% (n = 1) among American Indian/Alaskan Native women. Hence, there is a strong need to increase the number of ethnic/racial groups represented in BF studies in the U.S. and conduct studies that look at a single ethnic/racial group at a time, especially American Indian/Alaskan Native women.

Consistent with the global evidence [118], MCPs were found to impact BF outcomes among minority women in the U.S. There is now a need to conduct implementation science research [119] to understand how best to scale up and sustain the Baby Friendly Initiative in geographical areas serving ethnic/racial minority women [120, 121] . Given the negative impact that cultural barriers can have on infant feeding behaviors and maternal and child health outcomes [20], such studies need to identify the best approaches to implement effective maternity staff trainings that include a culturally sensitive communication skills building component so as to avoid stereotyping BF behaviors in different ethnic/racial groups [20].

It is notable that we did not identify any study focusing on behavior change social marketing campaigns targeting minority women in the U.S. The persistence of this previously documented gap [120, 122] needs to be urgently addressed given how important these campaigns, in combination with other strategies, have been for improved breastfeeding outcomes within other countries [122].

The great majority of studies were graded as being very low or low quality. Future research in this area needs to develop more robust studies. It is important that funding agencies such as NIH and CDC allocate more funding for understanding how best to protect, promote, and support BF among minority women in the US.

Lastly, no single study formally tested the combined impact of interventions to improve BF outcomes among minority women across different levels of the socioecological model from a multisectoral perspective. The U.S. could benefit from the global experience with evidence-informed policy toolboxes that can guide the development of sound BF protection and promotion, and support research agendas, policies, and programs based on complex adaptive systems frameworks such as the Breastfeeding Gear Model (see Table 1) [31, 123, 124] . Specifically, it will be important for a multisectoral committee to assess the strengths and weaknesses of each of the key enabling environments needed to improve BF outcomes among minority women in the United States. These include evidence-based advocacy with a strong equity lens, political will, legislation and resources for maternity benefits, workforce training and implementation of high quality culturally sensitive facilities and community based BF support programs, culturally appropriated social marketing behavior change communication campaigns, implementation research, and decentralized coordination from the national to the local level [31]. Finding from such assessments can then help inform the design of BF policies and implementation and evaluation of large scale interventions for minority women based on sound human rights and health economics frameworks [26–28].

## Conclusions

In conclusion, our SR found that policy and community level interventions at the WIC, health facility, and community agency level are likely to improve BF outcomes. Multi-point interventions that include prenatal, perinatal, and postnatal components including peer counseling are very promising. However, combining interventions at different levels of the socioecological model has not been studied in the U.S. Thus, it is strongly recommended that large scale implementation research that addresses the different health and social environments necessary for successful BF [27] be the next BF research frontier to explore ways to strengthen BF protection, promotion and support among minority women in the U.S.

## Supplementary Information

**Additional file 1.** Online Supplementary Material 1. MESH terms used in Systematic Review.

**Additional file 2.** Studies' data extraction information.

## Data Availability

All data generated or analyzed during this study are included in this article and supplementary files.

## Abbreviations

BF: Breastfeeding
SR: Systematic Review
PRESS: Peer Review Electronic Search Strategies
U.S.: United States of America
HP: Healthy People Initiative
HP2020: Healthy People 2020
EBF: Exclusive BF
CDC: The Centers for Disease Control
NIS: National Immunization Survey
ACA: Affordable Care Act
PRAMS: Pregnancy Risk Assessment Monitoring System
AA: African-American
AI/AN: American Indian/Alaska Natives
WIC: The Special Supplemental Nutrition Program for Women, Infants and Children
NHANES: National Health and Nutrition Examination Survey
NIH: National Institute of Health
CPM: Centering Pregnancy Model
BFH: Baby Friendly Hospital
BFHI: Baby Friendly Hospital Initiative
SSC: Skin-to-Skin Contact
PC: Peer Counselor
SID: Sudden Infant Death Syndrome
MCP: Maternal Care Practices
StEP: Starting Early Program
HSP: Healthy Start Program
CHAMPS: Communities and Hospitals Advancing Maternity Practices
LATCH: Lactation Advice Through Texting Can Help
PRISMA: Preferred Reporting Items for Systematic Reviews and Meta-analyses
GRADE: Grades of Recommendation, Assessment, Development, and Evaluation
BFGM: Breastfeeding Gear Model
RCT: Randomized Controlled Trial
CHAMPS: Communities and Hospitals Advancing Maternity Practices initiative
NFN: Nurturing Family Network
IBCLC: Certified Lactation Consultant
CLC: Certified Lactation Counselor
