# Supplementary material for: Impact of breastfeeding interventions among United States minority women on breastfeeding outcomes: a systematic review
Source: Int J Equity Health. 2021 Mar 6;20:72. doi: 10.1186/s12939-021-01388-4 (PMC7936442; doi:10.1186/s12939-021-01388-4)
Supplement: Supplementary file 1 — Additional file 1. Online Supplementary Material 1. MESH terms used in Systematic Review. [file 12939_2021_1388_MOESM1_ESM.docx]

**Online Supplementary Material 1. MESH terms used in Systematic Review**

**Ovid MEDLINE(R) ALL**

**Searched from inception on April 17, 2020**

| 1 | [breastfeeding concept] |
| --- | --- |
| 2 | (infant feeding* or breastfe* or breast-fe* or bottle fe* or bottlefe* or infant formula* or breastmilk or breast milk or wean or weaned or weaning).mp. |
| 3 | ((feeding or fed or food or foods) adj2 (solid or solids or formula or baby or babies or infant or infants or infancy)).mp. |
| 4 | lactation.mp. |
| 5 | breast pump*.mp. |
| 6 | Infant Formula/ or milk substitutes/ or infant food/ |
| 7 | Milk, Human/ |
| 8 | exp Infant Nutritional Physiological Phenomena/ |
| 9 | responsive feeding.mp. |
| 10 | room* in.mp. |
| 11 | or/2-10 |
| 12 | 11 not (exp geographic locations/ not (cities/ or islands/ or "oceans and seas"/ or exp united states/ or puerto rico/ or guam/ or american samoa/ or United States Virgin Islands/)) |
| 13 | limit 12 to yr="2009 -Current" |
| 14 | 13 not (animals not humans).sh. |
| 15 | [minority population concept] |
| 16 | Minority groups/ |
| 17 | Minority health/ |
| 18 | exp ethnic groups/ |
| 19 | continental population groups/ or exp african continental ancestry group/ or exp american native continental ancestry group/ or exp asian continental ancestry group/ or oceanic ancestry group/ |
| 20 | (african* or black* or hispanic* or latino* or latina* or latinx or puerto ric* or american indian* or native american* or asian* or chinese or filipino* or filipina* or indian* or korean* or japanese or vietnamese or pacific island* or hawaiian* or hawai'ian* or american samoa* or guam* or fiji* or marshall* or tonga* or mariana* or caribbean* or jamaica* or haitian* or mexican* or hmong or laotian*).mp. |
| 21 | (minorit* or underrepresented or under-represented or underserved or under-served or ethnic* or race or races or racial*).mp. |
| 22 | or/16-21 |
| 23 | [low income population] |
| 24 | (SES or socioeconomic or socio-economic).mp. |
| 25 | income*.mp. |
| 26 | financ*.mp. |
| 27 | (temporary assistance for needy families or tanf or welfare or (tax$ adj4 credit$) or public assistance or welfare or aid to families with dependent children or medicaid recipient*).mp. |
| 28 | (poverty or poor).mp. |
| 29 | (low SES or low socioeconomic or low socio-economic).mp. |
| 30 | income/ |
| 31 | social welfare/ |
| 32 | financial support/ |
| 33 | public assistance/ |
| 34 | financing, government/ |
| 35 | maternal welfare/ |
| 36 | food assistance/ |
| 37 | aid to families with dependent children/ |
| 38 | medical assistance/ or medicaid/ |
| 39 | exp socioeconomic factors/ |
| 40 | or/24-39 |
| 41 | 22 or 40 |
| 42 | 14 and 41 |
